# Dissemination and implementation science for global ophthalmology: closing the last-mile translational gap

**DOI:** 10.3389/fpubh.2026.1863810

**Published:** 2026-06-19

**Authors:** Ana Bastos de Carvalho, S. Lee Ware, Christina R. Studts, Wanjiku Mathenge

**Affiliations:** 1 Department of Ophthalmology and Visual Sciences, University of Kentucky College of Medicine, Lexington, KY, United States; 2 Adult & Child Center for Health Outcomes Research & Delivery Science (ACCORDS), University of Colorado School of Medicine, Aurora, CO, United States; 3 Department of Pediatrics, University of Colorado School of Medicine, Aurora, CO, United States; 4 Rwanda International Institute of Ophthalmology School of Ophthalmology, Kigali, Rwanda

**Keywords:** artificial intelligence, blindness, gender equity, global health, health disparate minority and vulnerable populations, implementation science, low vision, ophthalmology

## Abstract

Despite remarkable advances in ophthalmic diagnostics and therapeutics over the past decade, the global prevalence of avoidable visual impairment and blindness has remained unchanged. This paradox reflects a crisis of care delivery and the persistent failure to consistently, equitably, and effectively translate evidence-based interventions into routine practice for the millions who need them most. Dissemination and implementation (D&I) science offers ophthalmology rigorous, evidence-informed frameworks to close this last-mile translational gap. Drawing on the established body of D&I science theory, methods, and applications, this review argues that ophthalmology has been slow to harness these tools, and that doing so now is imperative. We examine the current evidence for D&I science applications across key domains of global eye care: equitable care delivery in the US, global eye health in low- and middle-SDI regions, gender equity, and the implementation of artificial intelligence-based diagnostic tools. Across each domain, we identify both promising examples of D&I science in action and critical gaps in evidence, equity, and sustainability. Achieving vision health equity worldwide will require ophthalmology to treat the dissemination, implementation, and sustainment of evidence-based eye care as central to its mission as the development of the innovations themselves.

## Highlights

- The global prevalence of avoidable visual impairment has remained unchanged over the past decade, reflecting a crisis of care delivery rather than a gap in therapeutic discovery.
- Dissemination and implementation (D&I) science provides rigorous, evidence-based frameworks to close the last-mile translational gap in eye care
- Disparities in eye care disproportionately affect women, racial and ethnic minorities, low-income populations, and residents of low/middle-SDI regions worldwide. D&I science offers targeted tools to characterize and disassemble these structural barriers.
- Proven D&I strategies have demonstrated success in improving equitable access to eye care but remain underutilized and understudied.
- Emerging technologies such as AI-based diagnostic tools hold promise for expanding access in underserved regions but will only fulfil their potential if developed with diverse, locally sourced datasets and deployed using thoughtful implementation strategies that address infrastructure, workforce, and equity barriers.

## Introduction

### The last-mile translational gap: implementation

Over the past decade the global prevalence of avoidable visual impairment and blindness in adults aged 50 years and older has remained unchanged (1) despite many advances in diagnostics and treatment for blinding diseases. In the United States (US) alone, many millions suffer visual impairment or blindness due to an intervention-amenable cause (2–5). Most people losing their sight would gain more from delivery of already-discovered interventions than from new discoveries, but access is far from universal. Ophthalmology faces a crisis of care delivery, not a shortfall of therapeutic discovery. The larger task is to consistently, effectively, and equitably deliver evidence-based eye care to millions of underserved people in the US and worldwide.

Oft-cited reports described research pipeline lag times of 17–20 years separating innovation from practice in routine care settings (6, 7). More recent work has suggested that this lag may be even longer (8). Further, up to two-thirds of guideline-reinforced clinical innovations may never be adopted (9). For those adopted, interventions often fall short of trial-level efficacy in real-world settings (10). For millions facing vision loss, inequities in diagnosis, treatment, or access to care place the most effective screenings, services, and therapies out of reach (11, 12). Recent scholarship has challenged the precision of the 17-year figure, arguing that the time from evidence to practice varies substantially by clinical context — but that the core problem it represents, unequal access to the benefits of scientific progress, remains as urgent as ever and is best understood as a matter of health equity (13).

In parallel with the maturation of bioscience research and the clinical medicine pipeline, (14) the systemic failure to deliver evidence-based care has been referred to as the evidence-to-practice gap, the second translational gap, (15) and as gaps in T2, (16) T3, (17, 18) and T4 translation (19). While T1 research moves discoveries to patients, T2–T4 focus on ensuring treatments reach the general public and improve health at scale, and it is here that the system most often fails. Dissemination and implementation (D&I) science — the body of knowledge relating to techniques of successful transmission, installation, delivery, and maintenance of evidence-based clinical practices (20) — is the underused tool by which ophthalmology can close this last-mile translational gap, answering: how do we reach everyone who needs our services, and how do we consistently deliver evidence-based care to everyone we reach?

This manuscript was written as an opinion paper and narrative review. The literature cited was selected based on the authors' expertise across D&I science, global ophthalmology, and health equity research, with the aim of illustrating key themes and providing representative examples rather than offering an exhaustive or systematic synthesis of the available evidence.

### Dissemination and implementation science in healthcare

As awareness of the evidence-to-practice gap grew during the 1990s, the aims of clinical research were broadened beyond the establishment of internal validity (efficacy trials) to attend to questions of external validity (effectiveness trials) (21). When demonstrations of effectiveness still failed to ensure broad adoption, the D&I science of healthcare delivery was called for, (22, 23) drawing on decades of research (24) into how innovations spread (22).

Over the past decade, D&I science has matured theoretically and methodologically, evolving from a focus on the barriers preventing innovation adoption to an emphasis on the strategies by which innovation may be disseminated, implemented, equitably delivered, and sustained through a balance of fidelity and adaptations to changing context (25, 26). These implementation strategies (27) may involve any or many levels of the socioecological model (e.g., patient, practitioner, organization, system, policy) and may be applied to promote the adoption, delivery, and maintenance of innovations across the care continuum (26). More than anything, D&I science is about how to successfully manage change. It provides the tools with which eye care researchers can translate efficacious practices into effective routine care for all who could benefit (28).

Key successes illustrate D&I science's contributions (29). Patient safety checklists dramatically reduced catheter infections only when supported by organization level implementation strategies (30, 31). The Diabetes Prevention Program (DPP) achieved nationwide adoption and fiscal self-sustainment through CDC support and RE-AIM framework evaluation (32, 33). And the CDC's packaging of evidence-based HIV prevention interventions alongside implementation strategies increased uptake among more than 11,000 agencies.(34, 35).

Emmons and Colditz estimated that better D&I of evidence-based practices might have prevented more than half of 21st century cancers to date (36). With historically strong contributions from primary care, oncology, mental health, and chronic disease management, D&I science has now been entered the research agenda of numerous specialties, (37) with independent pressures – licensure requirements, funding priorities, accreditation standards, and payer pressures – increasing its relevance for clinicians, researchers, and care organizations (38).

D&I methods such as hybrid implementation-effectiveness trials enable simultaneous collection of stakeholder feedback and clinical outcomes data to drive iterative improvements (39, 40). By generating generalizable data on implementation determinants and outcomes, D&I science empowers ophthalmologists to transfer evidence-based practices into routine care, close reach and consistency care gaps, and build learning health care systems (41, 42).

## D&I science for equitable eye care in the US

Evidence of insufficient and inequitable eye care in the US has steadily accumulated (43). Disparities disproportionately harm women, (44) racial and ethnic minorities, (45) indigenous groups, (46) the socioeconomically disadvantaged, (47–49) the un- and under-insured, (50) and rural populations (51). Systematic measures of eye disease and care gaps in the US are absent, (52) outdated, methodologically flawed, (53) or too narrow in scope (54–56). But based on the existing literature, US Black adults have had twice the visual impairment and blindness rates of non-Hispanic white adults, (57) mostly stemming from preventable causes (58). Black adults experience a three-times greater risk of vision-threatening diabetic retinopathy (59) and macular edema,(60) and five-times greater odds of unoperated cataracts (57, 61). Disturbingly, however outdated these observations, they constitute our most current knowledge.

The degree to which policy and health system changes over recent decades have closed these care gaps is urgent and open (62, 63). For example, Healthy People 2030 set a goal to increase DR screening from 64.8 to 70.3% by 2030 (64). Yet, six years on, screening rates show little detectable change and the unscreened population is not demographically representative (45).

D&I science provides tools to characterize and resolve breakdowns in equitable eye care delivery (65, 66). Ophthalmologist-led teams have applied it to primary care-based telemedicine diabetic retinopathy screening (TDRS) programs (67–71). Though several such programs have been established, their results vary widely and benefits are often not sustained (72–75). This variability is not incidental: it reflects the absence of systematic attention to the implementation determinants, such as organizational readiness, workflow fit, reimbursement structures, and staff buy-in, that predict whether a program will be adopted and maintained. By applying D&I theory and methods, we have identified and ranked key determinants of successful TDRS implementation,(76) enabling targeted strategies to promote facilitators and reduce barriers in current programs, and to design future ones with core dissemination, implementation, sustainment, and equity principles (76, 77). For TDRS programs to achieve sustained impact at scale, future efforts must move beyond demonstrating clinical efficacy and invest in designing for sustainability, including integration into existing workflows and billing infrastructure, and establishing quality monitoring systems that can detect implementation drift over time.

Glaucoma, another main cause of avoidable visual impairment in the US, presents similar opportunities. D&I science frameworks such as RE-AIM have been used for evaluation of community glaucoma programs, (78) and strategies including patient glaucoma education can improve attitudes towards pursuing care (79). Zhao et al described strategies that improved screening and follow-up adherence for Black individuals in a low-income urban setting, including shortening time to scheduled follow-up, patient testimonials, patient education, and provision of vouchers stating the monetary value of the free exam (80). Similar strategies have expanded access to eye care for adults (81, 82) and children, (83, 84) though implementation tailoring is crucial. Comparable interventions may fail in diverse settings or populations, (85) highlighting the importance of D&I science in global ophthalmology.

## D&I science for equitable eye care worldwide

D&I science may have an even larger impact in lower socio-demographic index (SDI) regions, (86) where avoidable visual impairment remains disproportionately high (87). Rwanda's Primary Eye Care (PEC) program, developed through policy change, advocacy, robust D&I protocols, and ongoing monitoring (88–90). The PEC program transformed the landscape of eye health in Rwanda over a decade through strategies targeting workforce capacity, supply chains, infrastructure, and affordability (91). It leveraged contextual factors, including perceived links between vision and national development, beliefs in health as a human right, community-based health insurance, and leaders' trust in the rapid assessment of avoidable blindness national survey data. The PEC program owes its sustainability to its deliberate integration into national health policy and government financing structures. This feature distinguishes it from many global eye health initiatives that have collapsed following withdrawal of external donor funding. Whether the PEC model is scalable to other low-SDI contexts, however, remains to be determined, as its success was shaped by Rwanda-specific contextual enablers that may not be present or reproducible elsewhere. Implementation research testing this program's transferability to other contexts is needed but is lacking.

Overall, however, D&I science application in low SDI regions remains limited. Cataracts are the first or second largest cause of blindness in most such regions (1). Yet, a Cochrane systematic review screened nearly 3,000 studies on implementation strategies for cataract surgery access, and identified only two eligible trials, neither of which measured change in cataract blindness prevalence or assessed equity outcomes across population subgroups (92). A subsequent broader systematic review similarly found insufficient evidence to recommend specific implementation strategies in such settings (93). Meanwhile, population-level data confirm a severe access gap: effective cataract surgical coverage in low-income countries remains below 15%, compared to over 60% in high-income settings (94). This gap persists despite effective surgical techniques, because the implementation infrastructure needed to deliver them at scale is inadequate and understudied. Assessment of barriers, facilitators and implementation strategies to optimize trained workforce, affordable supply chains, demand generation, and quality assurance system is needed, but the absence of rigorous implementation trials in this space means that even where cataract programs exist, we know little about what drives their quality and long-term sustainability.

Similarly, there is a dearth of evidence on implementation strategies for spectacle and compliance, the main cause of visual impairment in low SDI nations. Effective refractive error coverage in low-income countries is as low as 28.3%, (95) with even lower rates in females, older adults, and remote populations. Strategies such as patient education and free spectacles for children seem effective (96). but these approaches rely on financial investment, often unavailable in low/middle SDI countries. Donors contributions may not be sustainable and can decrease unexpectedly in response to global events and policy changes, (97) ending access to interventions. This donor dependency is a structural vulnerability that D&I science can address. Implementation frameworks that explicitly plan for financing sustainability, task-shifting to lower-cost community health workers, and integration into existing primary care structures offer a more durable path than project-based donor models (98). Further research should combine quick low-cost refraction by community health workers or teachers, affordable high-quality spectacles, and education on spectacle benefits.

## D&I science for gender equity in global eye care

Gender equity represents one of the most under-addressed disparities in global eye health. Women bear a higher burden of blindness and vision loss than men, with 27% higher prevalence and 17% greater rate of years lived with disability (YLD), with the disparity most pronounced in older age groups and lower-resource settings (99). Of the estimated 253 million people with visual impairment worldwide, 55% are women, and multiple factors contribute to this imbalance (100). Women's greater life expectancy heightens susceptibility to age-related conditions; hormonal factors influence cataract formation; and women face larger barriers to accessing eye care. These aspects are compounded by disparities in education, economic independence, and healthcare accessibility (101).

These barriers are not uniformly distributed across the globe but take context-specific forms that must be understood to design effective implementation strategies. Family responsibilities leave women with limited time for self-care, while safety risks, social taboos, and often the requirement of a male escort, make independent travel to clinics difficult. Caring for children also exposes women to higher risk of trachoma, and smoke exposure in domestic cooking contributes to eye irritation (102). In sub-Saharan Africa, women in some settings must seek permission from a male household head to attend health clinics. In Southeast and East Asia, older women with vision loss are frequently socially excluded and become housebound (103). These deep structural and cultural barriers compound the already elevated biological risk that women face for blinding conditions.

The gap between women's need and their access to surgical services has been well documented. A systematic review and meta-analysis of 22 studies in India found that women had 69% higher odds of being blind due to cataract than men, while having 27% lower odds of receiving cataract surgery (104). While striking, these findings are largely drawn from South Asian contexts and may not be generalizable across the diverse settings in which gender inequities in eye care occur. This pattern is present in pediatric eye care as well: reports consistently show significantly more boys than girls receive surgery for congenital or developmental cataract, (105, 106) but gender inequity can be avoided if recognized.

Despite the scale of inequity, the evidence base for implementation strategies in global ophthalmology remains limited and of critically poor quality. Mercer et al. sought evidence for interventions to increase gender equity in eye care across low-resource settings and found only four RCTs and nine observational studies, all judged to have serious risk of bias. Six studies examined interventions involving training rural community volunteers to identify, educate, and assist individuals with eye care needs, and these were associated with reduced gender inequities in blindness, clinic attendance, cataract surgical coverage, and trachoma treatment coverage, albeit with low-to-very-low quality evidence (107). In short, despite documented disparity, the field has produced almost no rigorous evidence on how to address it.

There are, however, examples that can guide D&I strategies. Baruwa et al. assessed the impact of five years of free cataract testing and access to low-cost surgery in rural China. They found that willingness to pay for surgery increased from 50 to 91% among women, thereby reversing the gender gap that existed at baseline, when women were less willing to pay than men (108). This demonstrates that carefully designed, sustained implementation strategies targeting financial barriers and awareness can shift gender disparities in eye surgery. A broader scoping review of eye care interventions in low- and middle-income countries found that beyond knowledge improvements, gender-sensitive care, household power dynamics, gender-based discrimination, and cultural expectations all represent additional barriers that implementation programs must plan for. However, none of the studies reviewed directly addressed these social determinants (109).

The underrepresentation of women in eye health leadership further compounds the problem. Women make up 70% of the health workforce globally but hold only 25% of senior roles, and in the eye health sector specifically, only 28.3% of board members of eye health organizations are women (110). This imbalance limits the degree to which gender-responsive perspectives are incorporated into programs, policies, and resource allocation. Achieving gender equity in global eye care thus requires D&I science to work on two parallel tracks: designing and rigorously evaluating implementation strategies that address access barriers faced by women and girls; and building the gender-disaggregated evidence base needed to identify what works across different contexts.

## D&I science for artificial intelligence in global eye care

Artificial intelligence (AI) advances are rapidly changing the possibilities for ophthalmic screening and diagnosis in low-resource settings, but major implementation barriers remain. While the first AI algorithm for diabetic retinopathy screening was approved by the Food and Drug Administration (FDA) over eight years ago, access remains limited due to costs and contextual factors (111).

The majority of AI studies in ophthalmology report retrospective diagnostic accuracy metrics (sensitivity and specificity) under controlled conditions, but few have demonstrated benefit in prospective, real-world clinical settings or reported patient-level health outcomes (112). A scoping review of AI tools for diabetic retinopathy in low- and middle-income countries (LMICs) found that while reported diagnostic accuracies were generally high, the vast majority of studies focused on sensitivity and specificity, with limited information on cost, regulatory approvals, or whether AI use improved health outcomes. The review identified only three studies that focused on the implementation of an AI tool into an active clinical pathway, concluding that further research beyond these metrics is needed before wider implementation (113). Rigorous randomized controlled trials evaluating AI models in ophthalmology remain scarce, and current evidence is insufficient to establish whether AI interventions improve clinical outcomes (114).

Companies are developing AI tools for diagnosis and assessment of glaucoma, (115) age-related macular degeneration; (116) retinopathy of prematurity, and other pediatric retina diseases (117). Crucially, AI model performance is highly correlated with training data quality, yet many algorithms are trained on datasets from high-income countries and populations (118, 119). These may not reflect pathologies in other populations or world regions, potentially limiting use in low SDI regions or low-income US populations. Locally sourced datasets and diverse international databanks are necessary for cross-population validity (118, 119) Additionally, targeting access barriers will be essential, (120) with approaches to build local capacity by training health workers; integration into existing workflows; (121) and off-grid or edge-computing models for areas with intermittent connectivity (122).

AI could also be leveraged for equitable access to medical and implementation knowledge in SDI regions where resources limit access to paywall-protected journals (123). OpenEvidence's free model places state-of-the-art evidence at the fingertips of US healthcare professionals, but does not permit international users. Similar software, such as DrOracle.AI, require paid subscriptions, limiting their use in low-resource settings. Implementation of AI-based health solutions in low- and middle-SDI regions will require strategies targeting poor connectivity, data privacy, user training, and patient education resources for diagnoses delivered via software.

As AI tools inundate healthcare, the urgent priority must be a shift from development to rigorous evaluation: D&I methods are needed to assess risk, performance, and financial feasibility before widespread deployment. The Rwanda RAIDERS study demonstrated the power of AI to drive patient action, showing that AI-based screening results increased uptake of follow-up specialized care by over 30% (124) In India, AI-powered chest X-ray screening for tuberculosis has bridged the diagnostic gap by identifying cases at a fraction of the cost and time (125). Integrating AI capabilities with public health policies and advocacy is essential for adoption, implementation, and sustainment of such tools in low- and middle-SDI regions and in underserved populations within high-income nations.

## Discussion and conclusion

From refractive error and DR screening to chronic management of glaucoma and age-related macular degeneration, ophthalmology faces a crisis of care delivery. While D&I science methods are growing across medicine, substantially fewer studies have assessed the effects of implementation strategies in eye care. Barriers and facilitators to care, inequity in care delivery, and challenges with patient compliance and adherence are well-described (126) but studies of systematic D&I-informed strategies to address them remain limited.

To equitably close the last-mile translation gap driving vision-related morbidity in the US and globally, collective awareness, acknowledgment, and commitment are requisite initial steps. The robust armamentarium of evidence, tools, and insights from D&I science can and should be leveraged to confront these alarming yet surmountable challenges in eye care throughout the world.

## Data Availability

The original contributions presented in the study are included in the article/supplementary material, further inquiries can be directed to the corresponding author.
